# Modifiable determinants of central obesity among the rural black population in the DIMAMO HDSS, Limpopo, South Africa

**DOI:** 10.3389/fpubh.2023.1165662

**Published:** 2023-05-12

**Authors:** Cairo B. Ntimana, Solomon S. R. Choma

**Affiliations:** Department of Pathology, Faculty of Health Sciences, School of Medicine, University of Limpopo, Polokwane, South Africa

**Keywords:** central obesity, smoking, unemployed, marital status, educational status

## Abstract

**Background:**

Central obesity is a leading risk factor for cardiometabolic diseases, in which body fat accumulates to a particular extent, and may negatively impact on health. The prevalence of abdominal obesity has increased over the last 10 years and currently surpasses that of overall obesity. There is a scarcity of data on the determinants of central obesity, especially among populations residing in rural Africa. The aim of the present study was thus to determine sociodemographic and lifestyle factors that are associated with central obesity.

**Methods:**

This was a cross-sectional, retrospective study. The present study used secondary data from the AWI-Gen phase 1 study. The study comprised 791 participants, of which 242 were men and 549 were women aged 40 years and above. The participants were selected by convenient sampling. Data were analyzed using the Statistical Package for Social Sciences version 27. A comparison of proportions was performed using the chi-square test, while a comparison of means was performed using an unpaired Student *t*-test. The association between sociodemographic and lifestyle factors with central obesity was analyzed using bivariate correlation, partial correlation, and binary regression analysis, and the statistical significance was set at a *p*-value of <0.05.

**Results:**

The proportion of central obesity in the total population was 59.9%, and significantly more women were centrally obese (79.6 vs. 15.3%, *p* = <0.001) as compared to men. Married status correlated positively and significantly with central obesity in both bivariate and partial correlations. Moreover, binary logistic regression further confirmed the positive association between married status and central obesity. Single status correlated negatively and significantly with central obesity. The correlation remained unchanged even after controlling for age and gender. Binary logistic regression showed that unemployment correlated significantly with central obesity. The proportion of smokers was also significantly higher in participants without central obesity than in those with central obesity (87.2 vs. 34.0%, *p* = <0.001). Smoking correlated negatively and significantly with central obesity in bivariate and partial correlations. In addition, binary logistic regression further confirmed the negative association between smoking and central obesity.

**Conclusion:**

The present study shows that in this population, central obesity is determined by gender, unemployment, and marital status.

## 1. Introduction

In developing nations, central obesity has become a major public health issue (1). It is one of the main causes of metabolic syndrome and associated disorders. Globally, waist circumference (WC) is used as a parameter to determine central obesity (2). Men and women with waists larger than 120 cm and 110 cm, respectively, are at very high risk of suffering from obesity-related health complications (2). Central obesity is a medical disorder in which body fat accumulates to a particular extent and may negatively impact on health, consequently shortening life expectancy and degrading health (3). With increasing rates in both adults and children, central obesity is currently the most preventable cause of mortality (3, 4). Over the past decade, abdominal obesity has increased even more rapidly than BMI (5, 6).

The estimated total prevalence of central obesity worldwide was 41.5%, with a significant increase from 31.3% in the 1990s (1985–1999) to 48.3% (2010–2014) (5). With rates of 42.4% in men and 61.3% in women, the prevalence of abdominal obesity has increased over the last 10 years and currently surpasses that of overall obesity (7). In South Africa, the prevalence of obesity was reported to be 67.0% (8). Moreover, in Limpopo, the proportion of waist circumference was found to be 34.6% of the total population, with women having a higher proportion than males (49.8 vs. 34.6%) (9).

Socioeconomic status (marital status, employment status, and the highest level of education), excessive dietary energy consumption, little or no physical activity, hereditary vulnerability, and lifestyle (smoking and alcohol consumption) have been reported to be associated with the increased deposition of fats in the fatty tissues, which can result in increased levels of fats in the visceral adipose tissues, subcutaneous adipose tissues, and increase in waist circumference (10–13). Alcohol intake is directly linked to waist circumference and a higher risk of abdominal obesity in men, but not in women (14). The prevalence of central obesity has been reported to be increasing worldwide, with the growth being higher in developing countries including South Africa. However, among these developing countries, there is little research on the relationship between socio-demographic and lifestyle factors and central obesity (15), hence the study aims to determine which sociodemographic factors and lifestyle factors are associated with central obesity.

## 2. Methodology

### 2.1. Study design, study population, and sampling

This population-based cross-sectional study is a retrospective study using AWI-Gen phase 1 data. The AWI-Gen phase 1 project selected its participants using random sampling. The AWI-Gen phase 1 database consisted of 1,399 participants aged above 40, and all participants were selected for the current study. Participants with incomplete records relevant to the current study were excluded, which resulted in a total sample size of 791 participants, of which 242 were men and 549 were women. All individuals gave their informed consent, and the Turfloop Research Ethics Committee (TREC) approved the study's protocol (TREC/264/2021:PG). The Dikgale Tribal Authority authorized the study's execution and provided written informed consent.

### 2.2. Measurement

An AWi-Gen questionnaire was used to obtain information from participants on age, gender, and socioeconomic factors. Body weight and height were measured using standard protocols with participants wearing light clothing without shoes. Obesity was diagnosed by measuring body mass index (BMI), which is weight in kilograms divided by the square of the height in meters. Individuals with a body mass index of ≥30 kg/m^2^ were considered obese. The optimal cutoff values for waist circumference (WC) were 94 cm in men and 80 cm in women. Central obesity was defined as a WC of ≥80 cm for women and ≥94 cm for men. Subcutaneous adipose tissues (SATs) and visceral adipose tissues (VATs) were measured using logic e ultrasound. The optimal cutoff values for VAT were 6.5 and 5.0 cm for men and women, respectively. The subcutaneous adipose tissue cutoff value was 1.82 cm in men and 1.46 cm in women. Individuals with a VAT of above 6.5 and 5.0 cm for men and women, respectively, were considered centrally obese by high VAT. Individuals with an SAT of above 1.82 and 1.46cm for men and women, respectively, were considered centrally obese by high SAT.

### 2.3. Statistical analyses

Data were analyzed using Statistical Package for Social Sciences (SPSS) version 27.0. Data were reported as frequency and percentages. A comparison of proportions was performed using the chi-square test, while a comparison of means was performed using an unpaired Student's *t*-test. The association between sociodemographic and lifestyle factors with central obesity was analyzed using bivariate correlation, partial correlation, and bivariate regression analyses. The statistical significance was set at a *p*-value of < 0.05.

## 3. Results

The mean age of the participants was 52.47 ± 8.24, and there was no significant difference in mean age between the women and men (Table 1). The proportion of widowed participants in the population was 19.4% and significantly more women were widowed as compared to men (22.5 vs. 11.3%, *p* = 0,021). Significantly more men were single, married, and divorced as compared to women. The proportion of unemployment in the population was 68.7%. Significantly more women were unemployed (49.2 vs. 19.5%, *p* = 0.05) compared to men. The proportion of alcohol consumption in the population was 33.7%, and significantly more men were alcohol consumers (78.5 vs. 16.5%, *p* = < 0.001) compared to women. The proportion of smoking in the population was 21.3%, and significantly more men were smokers (76.1 vs. 2.9%, *p* = < 0.001) compared to women. The waist circumference mean in the total population was 90.12 ± 16.07, and the waist circumference was significantly higher in women (94.36 ± 15.89, *p* = < 0.001) as compared to men (80.60 ± 11.83, *p* = < 0.001). The proportion of central obesity as measured by the high waist circumference, in the total population, was 59.9%, and significantly more women were centrally obese (79.6 vs. 15.3%, *p* = < 0.001) compared to men. In the total population, the SAT was 1.84±1.08. The SAT was significantly higher in women (2.24 ± 1.038, *p* = < 0.001) compared to men (0.94 ± 0.50, *p* = < 0.001). In the total population, the VAT was 6.56 ± 2.17 and significantly higher in women (6.78 ± 2.23, *p* = < 0.001) as compared to men (6.04 ± 1.96, *p* = < 0.001). The prevalence of high VAT in the total population was 61.3%, and significantly more women were centrally obese (82.5 vs. 17.5%, *p* = < 0.001). The proportion of high SAT in the total population was 55.0%, and significantly more women were centrally obese (96.0 vs. 4.0%, *p* = < 0.001).

**Table 1:** Characteristics of participants by gender.

**Socio-demographic status**				
	**Total (** ***n*** **)**	**Women**	**Men**	***P*** **-value**
*N*	791	549 (69.4 %)	242 (30.6 %)	
Age (years)	52.47 ± 8.24	52.48 ± 8.06	54.45 ± 8.64	0.952
**Marital status**				
Single % (*n*)	23.8% (165)	22.7% (113)	26.8% (52)	0.002
Married% (*n*)	53.5% (370)	52.4% (261)	56.2% (109)	
Divorced% (*n*)	3.3% (23)	2.4% (12)	5.7% (11)	
Widowed % (*n*)	19.4% (134)	22.5% (112)	11.3% (22)	
**Highest level of education**				
No formal education % (*n*)	9.2% (73)	10.2% (56)	7.0% (17)	0.144
Primary% (*n*)	35.3% (279)	35.0% (192)	36.0% (87)	
Secondary% (*n*)	52.9% (418)	52.9% (290)	52.9% (128)	
Tertiary% (*n*)	2.5% (20)	1.8% (10)	4.1 (10)	
**Employment status**				
Unemployed % (*n*)	68.7% (542)	49.2% (388)	19.5% (154)	0.05
**Life style**				
Smoking status % (*n*)	21.3% (149)	2.9% (14)	76.1% (134)	< 0.001
Alcohol consumption % (*n*)	33.7% (215)	16.5% (76)	78.5% (139)	< 0.001
**Anthropometric measurements and biochemical measurements**				
Waist circumference (cm)	90.12 ± 16.07	94.36 ± 15.89	80.60 ± 11.83	< 0.001
Central obesity (%)	59.9% (474)	79.6% (437)	15.3% (37)	< 0.001
Visceral adipose tissue (cm)	6.56 ± 2.17	6.78 ± 2.23	6.04 ± 1.96	< 0.001
High VAT	61.3% (485)	82.5% (400)	17.5% (85)	< 0.001
Subcutaneous adipose tissue (cm)	1.8 ± 1.07	2.21 ± 1.01	0.9 ± 0.52	< 0.001
High SAT	55.0% (482)	96.0% (411)	4.0% (17)	< 0.001

In the total study population, the proportion of central obesity was significantly higher in women than the proportion of men with central obesity (79.6 vs. 15.3%, *p* = < 0.001) (Table 2). The proportion of married and widowed participants was higher in those with central obesity compared to those without central obesity. The proportion of single and divorced participants was significantly higher in those without central obesity than in those with central obesity. The proportion of alcohol consumption was significantly higher in participants without central obesity than in central obesity (66.0 vs. 34.0%, *p* = < 0.001), and the proportion of smokers was also significantly higher in participants without central obesity than those with central obesity (87.2 vs. 34.0%, *p* = < 0.001).

**Table 2:** Comparison between socio-demographic profiles between participants with central obesity and those without central obesity.

**Characteristics**		**Without central obesity % (*n*)**	**With central obesity % (*n*)**	***P*-value**
Gender	Female	20.4% (112)	79.6% (437)	< 0.001
	Male	84.7% (205)	15.3% (37)	
Types of marital status	Single	25.7% (82)	17.5% (83)	0.006
	Married	36.6% (116)	53.6% (254)	< 0.001
	Divorced	4.7% (15)	1.7% (8)	0.017
	Widowed	13.2% (42)	19.4% (92)	0.026
Highest level of education	No formal education	38.4% (28)	61.6% (45)	0.803
	Primary	40.5% (113)	59.5% (166)	0.880
	Secondary	40.2% (168)	59.8% (250)	1.000
	Tertiary	40.0% (8)	60.0% (12)	1.000
Unemployment		40% (217)	60.0% (325)	1.000
Smokers		87.2% (130)	12.8% (19)	< 0.001
Alcohol consumption		66.0% (142)	34.0% (73)	< 0.001

Binary logistic regression of central obesity and sociodemographic statuses.

In bivariate correlation analyses, being single and divorced correlated negatively and significantly with central obesity. Married status correlated positively (*r* = 0.028) and significantly with central obesity. Smoking correlated negatively (*r* = −0.403) and significantly with central obesity (*p* = < 0.001). Alcohol consumers correlated negatively (*r* = −0.256) and significantly with central obesity (*p* = < 0.001). After controlling for both age and gender in partial correlation, being single correlated negatively (*r* = −0.128) and significantly with central obesity (*p* = < 0.001). Married status correlated positively (*r* = 0.218) and significantly with central obesity (*p* = < 0.001). There was no association between central obesity and the highest level of education. Smoking correlated negatively (*r* = −0.174) and significantly with central obesity (*p* = < 0.001).

**Figure 1:**
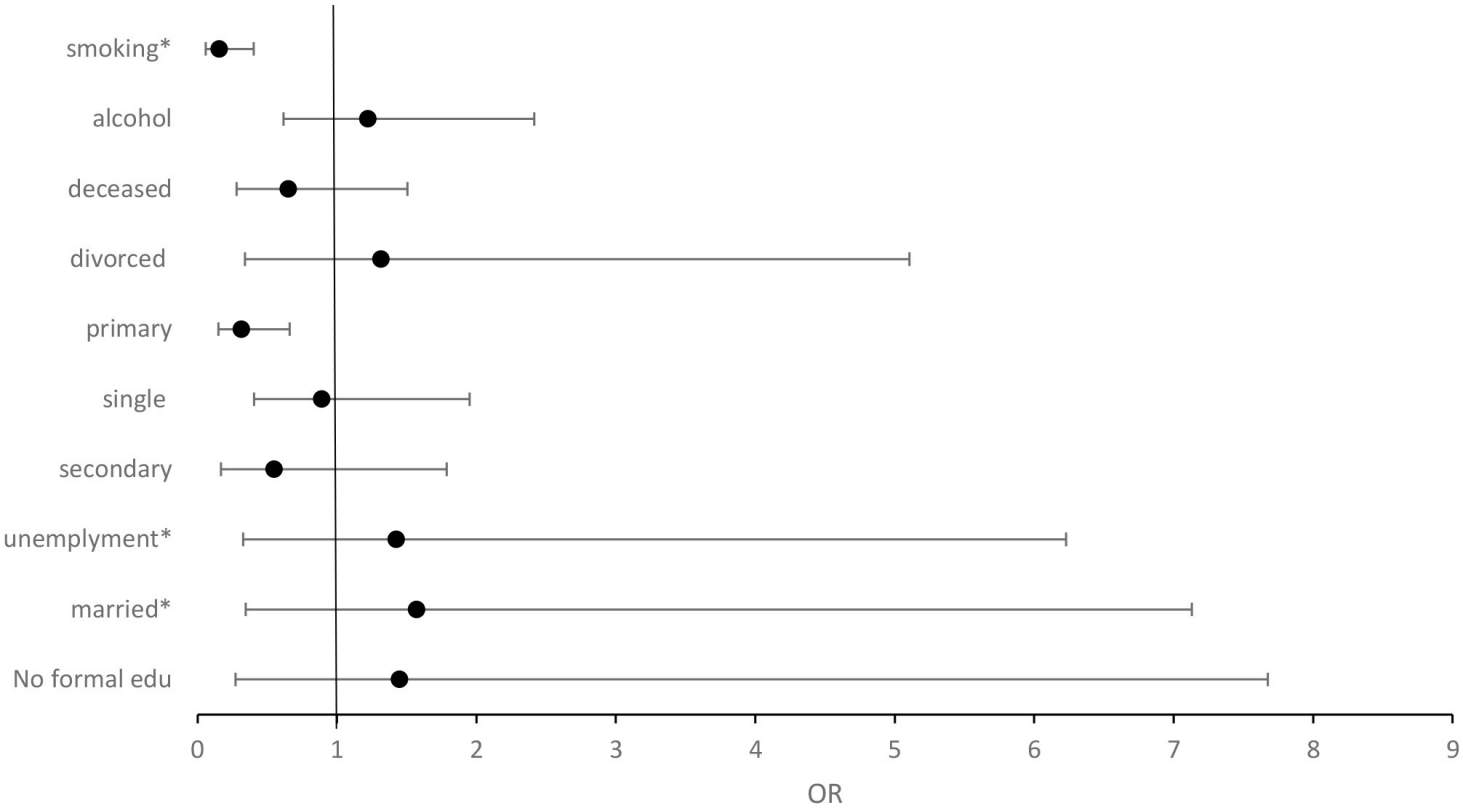
Forest plot illustrating the binary logistic regression of central obesity and sociodemographic factors. Normal = 1, OR>1 = positive relationship, OR < 1 = negative relationship. *P = <0.05.

Married participants were 1.571 times more likely to have central obesity (Figure 1). Unemployed participants were 1.425 times more likely to have central obesity. Smokers were 0.154 times less likely to have central obesity. There was no relationship between central obesity and alcohol consumption and the highest level of education.

## 4. Discussion

The present study consisted of more women (69.4%) as compared to men (30.6%); this may be because women are more likely to seek and use healthcare, have better health knowledge, adhere to medical programs, and ensure the health of others as well as their own (16). In addition, the majority of the men are either day workers in the area settings or have official jobs in urban areas, and hence are not able to take part in the study during the day (17). In the present study, there was no significant difference in single and married participants between women and men. Mashinya et al. (18) also reported similar findings. Significantly more women than men were divorced, and significantly more women than men had deceased partners. However, the findings of the present study are not in agreement with Mashinya et al. (18), who reported no significant difference in divorce and participants with deceased partners between men and women. The proportion of the unemployment rate in the population was 68.7%. Significantly more women were unemployed as compared to men. The findings of the present study are in agreement with Mashinya et al. (18), who reported a higher unemployment rate in women as compared to men. Unemployed individuals might not be able to afford nutritious food because of financial restrictions (19). The health of a person may be impacted by unhealthy or poor eating. One has an emotional emptiness in their life while they are unemployed. One frequently eats junk food and food rich in carbohydrates to fill boredom, which causes obesity (19).

The present study reported a proportion of alcohol consumption of 33.7% and men had a significantly higher proportion of alcohol consumption than women. These findings are in agreement with Maimela et al. (9), who also reported that significantly more male subjects were alcohol consumers as compared to female subjects. The findings of this study are similar to those of large community-based surveys conducted in other Sub-Saharan African countries (20). In Nigeria, 32.7% of men and 5.3% of women reported having consumed alcohol in the previous year (20). While in Tanzania, the rate of alcohol use was significantly higher in male participants at 38.5% as compared to female participants at 23.7% (21). Cultural norms dictate a double standard for the monitoring and punishment of deviance for girls and boys; this discrepancy between genders serves as a protective factor against risk-taking for female adolescents whereas boys have more freedom to interact with peers that teach and reinforce alcohol use (22–24). The main reason why men drink more than women is that traditionally the role of a woman is to be a mother and keeper of the house and family (24). Moreover, being drunk is considered unfeminine and does not resemble the image of women (24). Women alcoholics are said to be much uglier, disgusting, and abnormal, and they find it difficult to stop drinking even with medical assistance (24). The findings of the present study reported the proportion of smoking to be 21.3% and significantly more men were smokers compared to women. Maimela et al. (9) reported similar findings, and current smokers were significantly higher in men (29.2) as compared to women (4.5%). Cultural norms dictate that when a man smokes, it is just a health issue but when a woman smokes, it is considered a taboo (25, 26).

The waist circumference mean in the population was 90.12 ± 16.07, and women had a significantly higher waist circumference mean than men. Gaziano et al. (27), in their study of cardiometabolic risk in a population of older adults with multiple co-morbidities in rural South Africa, reported similar findings. One possible explanation for high WC in women is that most women available for the study were older and poor, forcing them to eat high-carbohydrate foods such as pap (18).

The present study found a higher proportion of central obesity (59.9%), with significantly more women than men having central obesity. The current study's findings are consistent with those of Liu et al. (28), who reported that the proportion of older adults with central obesity was significantly higher in both men and women. In addition, the proportion of central obesity was significantly higher in men as compared to women (29). In the present study, the proportion of single and divorced participants was significantly higher in those without central obesity than in those with central obesity, and the proportion of married participants was significantly higher in those with central obesity than those without central obesity. This is in agreement with Liu et al. (28) who reported similar findings. Bivariate correlation analysis confirmed that being single correlated negatively and significantly with central obesity. These correlations remain the same even after partial correlation analysis where age and gender were controlled. However, in binary logistic regression, single status is associated negatively but not significantly with central obesity. In other words, single participants were less likely to have central obesity. Memish et al. (30) reported similar findings.

Bivariate correlation analysis showed that married participants correlated positively and significantly with central obesity. These correlations remain the same even after partial correlation analysis where age and gender were controlled. Moreover, binary logistic regression further confirmed the positive relationship between married participants and central obesity. In agreement with the present study, several studies have indicated that married adults had a higher rate of central obesity than other marital status groups combined, and never-married people had a lower central obesity rate than married people (28, 30, 31). In the present study, married participants were more likely to be obese as compared to other marital statuses. This association may be explained by the fact that single, divorced, and participants with deceased partners experience psychosocial factors, such as social isolation, which may cause qualitative and quantitative changes in the amount of food consumed through loss of appetite, refusal to eat, or lack of motivation to prepare food, reducing the amount of energy consumed and, as a result, increasing the risk of having a poor nutritional status (32).

Binary logistic regression showed that unemployed participants were 1.425 times more likely to have central obesity. Unemployment has been reported to be associated with a higher waist circumference in men (33). Bakir et al. (31) reported an increase in waist circumference to be associated with unemployment. An unemployed individual might not be able to afford nutritious food because of financial restrictions (19). The health of a person may be impacted by unhealthy or poor eating. One has an emotional emptiness in their life while they are unemployed (19).

Bivariate correlation analysis showed that smokers were negatively and significantly associated with central obesity in the current study. The correlation remained unchanged even after controlling for age and gender in a partial correlation analysis. In addition, controlling for age, gender, and unknown confounders with binary logistic regression confirmed the negative relationship between smokers and central obesity. In agreement with the present study, several studies reported a negative association between smoking and central obesity (34–36). The mechanisms by which smoking leads to reduced waist circumference are complex and involve multiple neurochemical pathways. Most of the effects of smoking on body weight are mediated by nicotine inhaled from cigarette smoke (37). Nicotine increases the levels of various neurotransmitters, such as catecholamines, dopamine, and serotonin in the brain, which in turn suppresses appetite and consequently reduces food intake (37, 38). It is theoretically possible, but not yet confirmed, that nicotine has a negative effect on eating since is a drug that imitates the action of the neurotransmitter acetylcholine and can easily penetrate the blood–brain barrier (38). The inconsistencies between these studies may be that the present study used a lower sample size; another reason may be that the other study did not consider the marital status of the participants. There was no relationship between central obesity and alcohol consumption in the present study.

The present study reported central obesity to be associated with gender, being married, and being unemployed. However, in developed and developing countries, central obesity is associated with old age, gender, being employed, high socioeconomic status, smoking, and consumption of alcohol (39–46). The difference between the present study and studies conducted in developed and developing nations may be due to the difference in the geographical area, race, level of urbanization, and availability of food.

## 5. Study limitations

First, the findings of the study cannot be generalized to the entire population of Limpopo Province or the larger community outside of this group since the study used a convenient sampling method. Second, because the study was cross-sectional rather than longitudinal, causal relationships could not be assessed. Third, because alcohol consumption data were self-reported, researchers could not be certain about the effect of alcohol quantity on central obesity. Finally, because the smoking data were self-reported and the number of cigarettes smoked per day was not assessed, researchers could not be certain of the effect of smoking history and quantity on central obesity.

The present study recommends that similar studies be conducted, where there will be a random sampling of participants and measurements of the amount of alcohol consumed and the number of cigarettes smoked.

## 6. Conclusion

Central obesity correlated negatively with smoking, single status, and divorced status, and also correlated positively with married status and unemployed status.

## Data availability statement

The original contributions presented in the study are included in the article/supplementary material, further inquiries can be directed to the corresponding author.

## Ethics statement

The studies involving human participants were reviewed and approved by Turfloop Research Ethics Committee TREC/264/2021:PG. The patients/participants provided their written informed consent to participate in this study.

## Author contributions

Both authors listed have made a substantial, direct, and intellectual contribution to the work and approved it for publication.

## References

[1] Harbuwono DS Pramono LA Yunir E Subekti I. Obesity and central obesity in Indonesia: evidence from a national health survey. Med J Indones. (2018) 27:114–20. 10.13181/mji.v27i2.151233206709

[2] Ahmad N Adam SM Nawi A Hassan M Ghazi H. Abdominal obesity indicators: waist circumference or waist-to-hip ratio in Malaysian adults population. Int. J. Prev. Med. (2016) 7:82. 10.4103/2008-7802.183654 27330688 PMC4910307

[3] Zhang P Wang R Gao C Jiang L Lv X Song Y. Prevalence of central obesity among adults with normal bmi and its association with metabolic diseases in Northeast China. PLoS ONE. (2016) 11:e0160402. 10.1371/journal.pone.0160402 27467819 PMC4965061

[4] Zhang C Rexrode KM van Dam RM Li TY Hu FB. Abdominal obesity and the risk of all-cause, cardiovascular, and cancer mortality: sixteen years of follow-up in US women. Circulation. (2008) 117:1658–67. 10.1161/CIRCULATIONAHA.107.739714 18362231

[5] Wong MCS Huang J Wang J Chan PSF Lok V Chen X. Global, regional and time-trend prevalence of central obesity: a systematic review and meta-analysis of 13.2 million subjects. Eur J Epidemiol. (2020) 35:673–83. 10.1007/s10654-020-00650-3 32448986 PMC7387368

[6] Chen Y Peng Q Yang Y Zheng S Wang Y Lu W. The prevalence and increasing trends of overweight, general obesity, and abdominal obesity among Chinese adults: a repeated cross-sectional study. BMC Public Health. (2019) 19:1293. 10.1186/s12889-019-7633-0 31615464 PMC6794823

[7] Pou KM Massaro JM Hoffmann U Lieb K Vasan RS O'Donnell CJ. Patterns of abdominal fat distribution. Diabetes Care. (2009) 32:481–5. 10.2337/dc08-1359 19074995 PMC2646033

[8] Owolabi EO Ter Goon D Adeniyi OV. Central obesity and normal-weight central obesity among adults attending healthcare facilities in Buffalo City Metropolitan Municipality, South Africa: a cross-sectional study. J Health Popul Nutr. (2017) 36:54. 10.1186/s41043-017-0133-x 29282137 PMC5745975

[9] Maimela E Alberts M Modjadji SEP Choma SSR Dikotope SA Ntuli TS. The prevalence and determinants of chronic non-communicable disease risk factors amongst adults in the dikgale health demographic and surveillance system (HDSS) site, Limpopo Province of South Africa. PLoS ONE. (2016) 11:e0147926. 10.1371/journal.pone.0147926 26882033 PMC4755539

[10] Kim Y Jeong SM Yoo B Oh B Kang H-C. Associations of smoking with overall obesity, and central obesity: a cross-sectional study from the Korea National Health and Nutrition Examination Survey (2010-2013). Epidemiol Health. (2016) 38:e2016020. 10.4178/epih.e2016020 27221478 PMC4967909

[11] Philipsen A Hansen A-LS Jørgensen ME Brage S Carstensen B Sandbaek A. Associations of objectively measured physical activity and abdominal fat distribution. Med Sci Sports Exerc. (2015) 47:983–9. 10.1249/MSS.0000000000000504 25207926

[12] Martín AR Nieto JMM Ruiz JPN Jiménez LE. Overweight and obesity: the role of education, employment and income in Spanish adults. Appetite. (2008) 51:266–72. 10.1016/j.appet.2008.02.021 18406494

[13] Sarlio-Lähteenkorva S Silventoinen K Lahti-Koski M Laatikainen T Jousilahti P. Socio-economic status and abdominal obesity among Finnish adults from 1992 to (2002). Int J Obes. (2006) 30:1653–60. 10.1038/sj.ijo.0803319 16607386

[14] Uemura MY Ohira T Yasumura S Sakai A Takahashi A Hosoya M. Association between lifestyle habits and the prevalence of abdominal obesity after the Great East Japan Earthquake: the Fukushima Health Management Survey. J Epidemiol. (2022) 32:496–501. 10.2188/jea.JE20200597 33814507 PMC9551296

[15] Arabshahi S. Predictors of change in weight and waist circumference: 15-year longitudinal study in Australian adults. Eur J Clin Nutr. (2014) 68:309–15. 10.1038/ejcn.2013.260 24398635

[16] Osamor P Grady C. Womenand#39;s autonomy in health care decision-making in developing countries: a synthesis of the literature. Int J Womens Health. (2016) 8:191–202. 10.2147/IJWH.S105483 27354830 PMC4908934

[17] Van Zyl S Van der Merwe LJ Walsh CM Groenewald AJ Van Rooyen FC. Risk-factor profiles for chronic diseases of lifestyle and metabolic syndrome in an urban and rural setting in South Africa. Afr J Prim Health Care Fam Med. (2012) 4:1346. 10.4102/phcfm.v4i1.346

[18] Mashinya F Alberts M Cook I Ntuli S. Determinants of body mass index by gender in the Dikgale Health and Demographic Surveillance System site, South Africa. Glob Health Action. (2018) 11:1537613. 10.1080/16549716.2018.1537613 30392446 PMC6225484

[19] Herber G-C Ruijsbroek A Koopmanschap M Proper K van der Lucht F Boshuizen H. Single transitions and persistence of unemployment are associated with poor health outcomes. BMC Public Health. (2019) 19:740. 10.1186/s12889-019-7059-8 31196081 PMC6567908

[20] Gureje O Degenhardt L Olley B Uwakwe R Udofia O Wakil A. A descriptive epidemiology of substance use and substance use disorders in Nigeria during the early 21st century. Drug Alcohol Depend. (2007) 91:1–9. 10.1016/j.drugalcdep.2007.04.010 17570618

[21] Mbatia J Jenkins R Singleton N White B. Prevalence of alcohol consumption and hazardous drinking, tobacco and drug use in Urban Tanzania, and their associated risk factors. int J Environ Res Public Health. (2009) 6:1991–2006. 10.3390/ijerph6071991 19742167 PMC2738894

[22] Mafa P Makhubele JC Ananias JA Chilwalo BN Matlakala FK Rapholo SF. Alcohol consumption patterns: a gender comparative study among high school Youth in South Africa. Glob J Health Sci. (2019) 11:92. 10.5539/gjhs.v11n2p92

[23] Schulte MT Ramo D Brown SA. Gender differences in factors influencing alcohol use and drinking progression among adolescents. Clin Psychol Rev. (2009) 29:535–47. 10.1016/j.cpr.2009.06.003 19592147 PMC2756494

[24] Bobrova N West R Malyutina D Malyutina S Bobak M. Gender differences in drinking practices in middle aged and older Russians. Alcohol Alcohol. (2010) 45:573–80. 10.1093/alcalc/agq069 21075855 PMC3943390

[25] Tehrani H Mahdizadeh M Peyman N Gholian-Aval M Charoghchian Khorasani E Jafari A. Exploration factors on smoking among female adolescents based on the viewpoints of Iranian adolescent girls. BMC Womens Health. (2022) 22:203. 10.1186/s12905-022-01791-1 35650621 PMC9158312

[26] Başar D Öztürk S Aksoy SA. Gender differences in smoking behaviour: analysing the changes for the 2008-2014 period in Turkey. Sosyoekonomi. (2021) 29:107–26. 10.17233/sosyoekonomi.2021.02.06

[27] Gaziano TA Abrahams-Gessel S Gomez-Olive FX Wade A Crowther NJ Alam S. Cardiometabolic risk in a population of older adults with multiple co-morbidities in rural south africa: the HAALSI (Health and Aging in Africa: longitudinal studies of INDEPTH communities) study. BMC Public Health. (2017) 17:206. 10.1186/s12889-017-4117-y 28212629 PMC5314614

[28] Liu X Chen Y Boucher NL Rothberg AE. Prevalence and change of central obesity among US Asian adults: NHANES 2011–2014. BMC Public Health. (2017) 17:678. 10.1186/s12889-017-4689-6 28841875 PMC6389198

[29] Lv J Chen W Sun D Li S Millwood IY Smith M. Gender-specific association between tobacco smoking and central obesity among 0.5 million chinese people: the China kadoorie biobank study. PLoS ONE. (2015) 10:e0124586. 10.1371/journal.pone.0124586 25897789 PMC4405570

[30] Memish ZA El Bcheraoui C Tuffaha M Robinson M Daoud F Jaber S. Obesity and, associated factors, —, Kingdom of Saudi Arabia 2013. Prev Chronic Dis. (2014) 11:140236. 10.5888/pcd11.140236 25299980 PMC4193060

[31] Bakir MA Hammad K Mohammad L. Prevalence of obesity, central obesity, and associated socio-demographic variables in Syrian women using different anthropometric indicators. Anthropol Rev. (2017) 80:191–205. 10.1515/anre-2017-0013

[32] Davidson K Arber S Marshall H. Gender and food in later life: shifting roles and relationships. In: Food for the Ageing Population. Elsevier (2009). p. 110–27. 10.1533/9781845695484.1.110

[33] Pan F Tian J Cicuttini F Jones G. Metabolic syndrome and trajectory of knee pain in older adults. Osteoarthritis Cartilage. (2020) 28:45–52. 10.1016/j.joca.2019.05.030 31394191

[34] Gasperin LOF Neuberger M Tichy A Moshammer H. Cross-sectional association between cigarette smoking and abdominal obesity among Austrian bank employees. BMJ Open. (2014) 4:e004899. 10.1136/bmjopen-2014-004899 25079922 PMC4120441

[35] Al-Riyami AA Afifi MM. The relation of smoking to body mass index and central obesity among Omani male adults. Saudi Med. J. (2003) 24:875–80. 12939676

[36] Audrain-Mcgovern J Benowitz NL. Cigarette smoking, nicotine, and body weight. Clin Pharmacol Ther. (2011) 90:164–8. 10.1038/clpt.2011.105 21633341 PMC3195407

[37] Benowitz NL. Nicotine addiction. N Engl J Med. (2010) 362:2295–303. 10.1056/NEJMra0809890 20554984 PMC2928221

[38] Mackay DF Gray L Pell JP. Impact of smoking and smoking cessation on overweight and obesity: Scotland-wide, cross-sectional study on 40,036 participants. BMC Public Health. (2013) 13:348. 10.1186/1471-2458-13-348 23587253 PMC3636072

[39] Pan X-F Wang L Pan A. Epidemiology and determinants of obesity in China. Lancet Diabetes Endocrinol. (2021) 9:373–92. 10.1016/S2213-8587(21)00045-0 34022156

[40] Ahirwar R Mondal PR. Prevalence of obesity in India: a systematic review. Diabetes Metab Syndr Clin Res Rev. (2019) 13:318–21. 10.1016/j.dsx.2018.08.032 30641719

[41] Olatunbosun ST Kaufman JS Bella AF. Central obesity in Africans: anthropometric assessment of abdominal adiposity and its predictors in urban Nigerians. J Natl Med Assoc. (2018) 110, 519–27. 30129507 10.1016/j.jnma.2018.01.001

[42] Hu L Huang X You C Li J Hong K Li P. Prevalence of overweight, obesity, abdominal obesity and obesity-related risk factors in southern China. PLoS ONE. (2017) 12:e0183934. 10.1371/journal.pone.0183934 28910301 PMC5598943

[43] Agyemang C Boatemaa S Frempong GA Aikins A. Obesity in sub-saharan Africa. Metab Syndr Switz Springer Int Publ. (2016) 1–13.

[44] Yu C Shi Z Lv J Du H Qi L Guo Y. Major dietary patterns in relation to general and central obesity among Chinese adults. Nutrients. (2015) 7:5834–49. 10.3390/nu7075253 26184308 PMC4517030

[45] Addo PNO Nyarko KM Sackey SO Akweongo P Sarfo B. (2015). Prevalence of obesity and overweight and associated factors among financial institution workers in Accra Metropolis, Ghana: a cross sectional study. BMC Res. Notes 8, 599. 10.1186/s13104-015-1590-1 26499885 PMC4619450

[46] Chukwuonye II Chuku A Onyeonoro UU Okpechi IG Madukwe OO Umeizudike TI. Prevalence of abdominal obesity in Abia State, Nigeria: results of a population-based house-to-house survey. Diabetes Metab Syndr Obes Targets Ther. (2013) 285. 10.2147/dmso.s43545 23946664 PMC3738251

